# Characteristics and treatable traits of patients with chronic obstructive pulmonary disease (COPD) with and without paid employment

**DOI:** 10.1186/s12931-021-01736-6

**Published:** 2021-05-12

**Authors:** Peter A. Jacobsen, Alex J. van ’t Hul, Remco S. Djamin, Jeanine C. Antons, Marianne de Man, Ulla Møller Weinreich, Martijn A. Spruit, Daisy J. A. Janssen

**Affiliations:** 1 Department of Respiratory Diseases, Aalborg University Hospital, Mølleparkvej 4, 9100 Aalborg, Denmark; 2 The Clinical Institute, Aalborg University, Aalborg, Denmark; 3 Department of Respiratory Diseases, Radboud University Medical Center, Radboud Institute for Health Sciences, 6525 GA Nijmegen, The Netherlands; 4 Department of Respiratory Diseases, Amphia Hospital, 4818 CK Breda, The Netherlands; 5 Department of Respiratory Diseases, 5406 Uden, The Netherlands; 6 Department of Research and Development, Ciro, Horn The Netherlands; 7 Faculty of Health, Medicine and Life Sciences, Nutrim School of Nutrition and Translational Research in Metabolism, Maastricht University, Maastricht, The Netherlands; 8 Department of Respiratory Medicine, Maastricht University Medical Centre (MUMC+), Maastricht, The Netherlands; 9 Department of Health Services Research, Care and Public Health Research Institute, Faculty of Health, Medicine and Life Sciences, Maastricht University, Maastricht, The Netherlands

**Keywords:** Chronic Obstructive Pulmonary Disease (COPD), Occupation, Workforce connection

## Abstract

**Introduction:**

Patients with COPD are vulnerable to workforce detachment. Better knowledge of features associated with paid work loss might be of help to design and select appropriate interventions.

**Method:**

This cross-sectional study aimed to explore the presence of treatable traits in COPD patients without paid work. Patients with COPD below 65 years at first referral to a hospital-based patient clinic were included. Using binary logistic regression analysis, the relationship between paid work and the following characteristics was explored: low daily physical activity, exercise, active smoking, Medical Research Council dyspnea scale (MRC), poor nutritional status, exacerbations, and fatigue (checklist individual strength (CIS)). Variables were adjusted for age, sex, forced expiratory volume in 1 s (FEV 1), and education level.

**Results:**

In total, 191 patients (47.3%) were without paid work. The following treatable traits were related to not being in paid work: < 5000 steps/day (OR 2.36, 95% CI (1.52–3.68)), MRC ≥ 3 (OR 1.78, 95%CI (1.14–2.77)), CIS ≥ 36 points (OR 1.78, 95% CI (1.10–2.87)), six-minute walk distance (6MWD) < 70% of predicted (OR 2.62, 95% CI (1.69–4.06)), and ≥ 2 exacerbations per year (OR 1.80, 95% CI (1.12–2.92)). Significant differences were also seen in age (OR 1.06, 95% CI (1.02–1.10) per year), FEV 1% predicted (OR 0.98, 95% CI (0.97–1.00) per % predicted increase), and medium/high education level (OR 0.62, 95% CI (0.41–0.93)). When adjusting for all variables the only treatable trait that remained significant was 6MWD.

**Conclusion:**

Patients without paid work are more likely to have treatable traits with 6MWD revealing the most significant association.

**Supplementary Information:**

The online version contains supplementary material available at 10.1186/s12931-021-01736-6.

## Introduction

As the third leading cause of death in the world, Chronic Obstructive Pulmonary Disease (COPD) impacts millions of lives every day [1]. It has been estimated that 5–6% of adults in the Netherlands above the age of 40 have COPD, which comes with, apart from the increased risk of death, large impairments in patients daily living and quality of life (QoL) [2, 3]. Two studies conducted in European countries reported that approximately 60% of COPD patients below retirement age are outside the workforce [4, 5]. Unemployment in general is associated with an increased risk of all-cause mortality and for patients with COPD also with a lower health related quality of life (HRQoL) and poorer medical adherence [6–9]. The poor workforce connection comes with large public expenses in the form of increased public support expenses and decreased tax revenue [10, 11]. The benefits of maintaining the workforce connection are therefore both from a societal and an individual perspective highly important.

Different characteristics of patients outside the workforce have been examined in COPD. Older age, female sex, poorer prognostic score (Body mass index (BMI), airflow Obstruction, Dyspnea, Exercise (BODE score)), more severe dyspnea (modified Medical Research Council (mMRC)), increased airflow obstruction, lower educational level and work related exposure to vapors, gasses, dust or fumes have all been associated with a poorer workforce connection with some discrepancies in the literature regarding the relationship of airflow obstruction with workforce connection [5, 12–15]. In a review of work-related outcomes in patients with COPD, Rai et al. concluded that there is a need for studies examining modifiable aspects of the disease in order to improve interventions to increase workforce connection [13].

This study was therefore performed to examine differences in modifiable characteristics (treatable traits) of COPD in patients with and without paid work. We hypothesize that certain treatable traits are more prevalent in patients without paid work than in patients with paid work.

## Method

### Study setting

At the time of the study public retirement was available in the Netherlands for all people of 65 years and older. Disability retirement is available to patients below 65 years with either physical or mental impairments that impairs their ability to work.

### Study design

This study is a secondary analysis of the COPD *sTRAITosphere* study and uses an observational cross-sectional cohort study design [16]. Data was collected upon referral from the general practitioner to a pulmonologist for a hospital-based outpatient consultation, through a comprehensive diagnostic examination. The Research Ethics Committee of the Radboud University Medical Centre, Netherlands, approved the study. Due to the observational nature of the study and the provision of usual care, written informed consent was waived (ref: 2017/3597).

### Study population

**Fig. 1:**
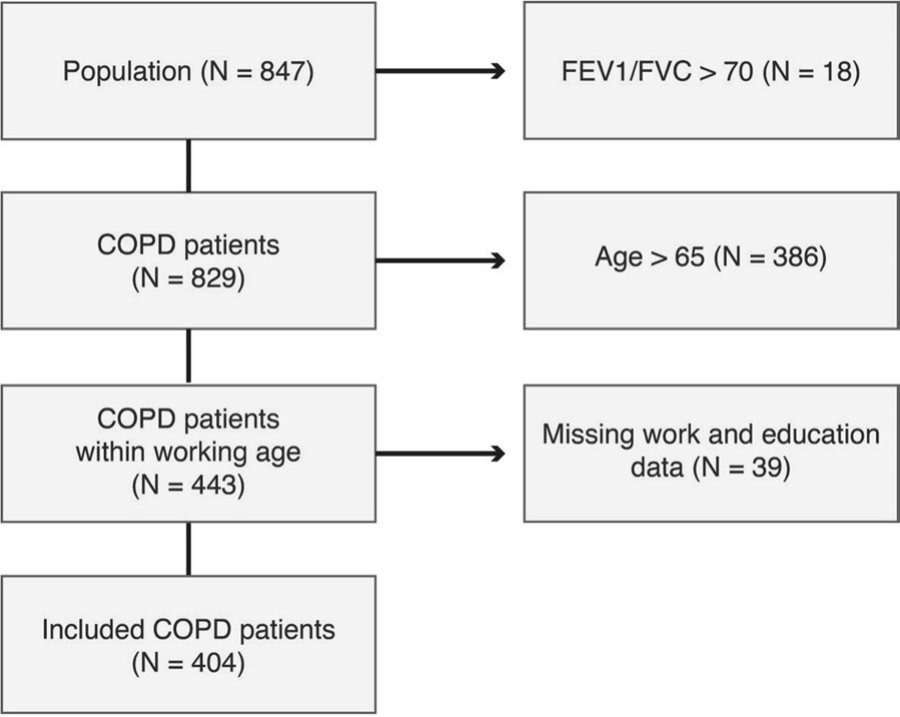
Flow chart of included patients. Abbreviations: Chronic obstructive pulmonary disease (COPD), forced expiratory volume in one second (FEV_1_), forced vital capacity (FVC)

Patients were included between April 2013 to December 2018. Included patients were the first time referred to a pulmonologist with a confirmed diagnosis of COPD and were free from exacerbations three months prior to inclusion. The diagnosis of COPD was confirmed using post-bronchodilator spirometry with a Tiffeneau-Penelli index < 70%. Data were collected from three centers in the Netherlands: Radboudumc, Nijmegen, Amphia hospital, Breda, and Bernhoven hospital, Uden. Patients aged 65 years and above were excluded as this was the minimum retirement age in the Netherlands during the study period (Fig. 1). Patients with incomplete data on education and work were also excluded.

### Outcomes and covariates

Patients reported at baseline examination if they were currently employed in paid work (“Yes” or “No”). Patients categorized as “No paid work” may therefore be unemployed or receive disability retirement.

The following other data were collected: Age, sex, comorbidities, educational level, spirometry (performed on Masterlab PFT; Vitalaire, Germany, spirometer, using post-bronchodilator (after inhalation of salbutamol 400 μg) spirometry estimated using the Global Lung Initiative equation), blood gas analysis, body mass index (BMI), six-minute walk test (6MWD), steps per day (recorded with activity monitor (Yamex or Dynaport)), exacerbation history during the last year (patients’ recall), checklist individual strength ((CIS), fatigue assessment), medical research council (MRC) score, clinical COPD questionnaire (CCQ) and smoking status. Education level was grouped into short (finished low-level secondary education or lower (International Standard Classification of Education(ISCED) level 0–2)), and medium/high (finished upper secondary education or above (ISCED 3–8)) [17].

The selection of treatable traits was chosen through the availability of evidence-based interventions, as done by van ‘t Hul et al. [16]. Treatable traits include smoking (currently smoking), activity-related dyspnea (MRC grade ≥ 3), frequent exacerbation (≥ 2 exacerbations per year), abnormal BMI (BMI < 21 or > 30 kg/m^2^), severe fatigue (CIS score ≥ 36 points), poor exercise capacity (6MWD < 70% predicted) and low daily physical activity (< 5000 steps per day) [18–24].

### Statistical analysis

Continues variables when normally distributed were reported using means and 95% confidence interval (CI), and when not normally distributed as medians with interquartile ranges. Normality was assessed using Shapiro-Wilks test, and visually assessed using histogram and Q-Q plot. Categorical variables were reported using counts and percentages. The distribution of treatable traits between workforce connection groups was shown using a bar chart.

Between group comparison in baseline data was done using an independent *T*-test for continuous data if normally distributed, and Wilcoxon signed rank test if not normally distributed. For categorical data with two groups chi-square test was used and for multiple groups ANOVA was used.

Primary outcome was examined using a between group comparison of workforce connection groups (paid work vs. not paid work) and treatable traits using binomial logistic regression, reporting odds ratios (OR) and 95% confidence intervals (CI). Age, sex, forced expiratory volume in 1 s (FEV_1_% predicted) and education level were examined together and included in the analysis of individual treatable trait to adjust for relevant confounders [13, 14, 25, 26]. Patients with missing data in individual treatable traits were excluded from the individual analysis. A multiple logistic regression model including all treatable traits with a *p*-value below 0.2 in univariate analyses was made to explore the importance of the individual treatable traits in relation to each other. Continuous variables were for the purpose of analysis with logistic regression checked for the linearity assumption by plotting the values against the corresponding logit of the model and if they were not linear then changed to relevant categories. Variables were checked for multicollinearity. Logistic regression models were checked for influential outliers defined as a Cook’s distance above 0.5 or a standardized residual larger than three. There were no influential outliers.

Analysis was reported using R version 1.3.1093. A p-value and confidence interval (CI) was performed for logistic regression with a two-tailed *p*-value of 0.05 considered significant.

## Results

## Population

The original database included 847 patients of which 404 (48%) patients were included in the current study (see Fig. 1). The median age in the study population was 58 (IQR 53–61) years, 60.6% had a low education level, and an average FEV_1_% of 56% predicted, corresponding to moderate airflow obstruction according to the Global initiative for Chronic Obstructive Pulmonary Disease (GOLD) classification [24]. A total of 191 (47%) of patients included did not have paid work (see Table 1).

**Table 1:** Demographics of population on workforce connection

Variable	Level	Total (n = 404)	Paid work (n = 213)	No paid work (n = 191)	*p*-value
Age (years)	Median [iqr]	58 [53, 61]	57 [52, 60]	58 [54.5, 62.0]	< 0.01
Age group	< 50 years	46 (11.4)	32 (15.0)	14 (7.3)	
	50–54 years	76 (18.8)	42 (19.7)	34 (17.8)	
	55–59 years	138 (34.2)	70 (32.9)	68 (35.6)	0.072
	60–64 years	144 (35.6)	69 (32.4)	75 (39.3)	
Sex	Female	211 (52.2)	103 (48.4)	108 (56.5)	0.122
Educational level	Low	245 (60.6)	118 (55.4)	127 (66.5)	
	Medium /High	159 (39.4)	95 (44.6)	64 (33.5)	0.039
Smoking status	Never smoker	9 (2.2)	6 (2.8)	3 (1.6)	
	Active smoker	214 (53.0)	104 (48.8)	110 (57.6)	
	Previous smoker	181 (44.8)	103 (48.4)	78 (40.8)	0.180
Smoking history (years)^π^	Median [iqr]	40 [30, 43]	38 [30, 40]	40 [32, 45]	0.085
FEV_1_% predicted	Mean (sd)	55.9 (18.8)	58.5 (18.5)	52.9 (18.8)	< 0.01
FVC% predicted	Mean (sd)	91.9 (17)	94.9 (17.2)	88.5 (16.2)	< 0.01
FEV_1_%/FVC%	Mean (sd)	0.5 (0.1)	0.5 (0.1)	0.5 (0.1)	0.151
FEV_1_% predicted	< 30	46 (11.4)	28 (13.1)	18 (9.4)	
	30–59	190 (47.0)	113 (53.1)	77 (40.3)	
	50–79	143 (35.4)	63 (29.6)	80 (41.9)	
	≥ 80	25 (6.2)	9 (4.2)	16 (8.4)	< 0.01
Height	Mean (sd)	170.1 (9.1)	171.4 (8.8)	168.7 (9.1)	< 0.01
Weight	Median [iqr]	71 [60, 85]	71 [61, 85]	71 [59, 86]	0.475
BMI (kg/m^2^)	Median [iqr]	24.8 [21.5, 28.6]	24.4 [21.8, 27.8]	25.3 [21.1, 29.0]	0.579
BMI	< 21 kg/m^2^	88 (21.8)	40 (18.8)	48 (25.1)	
	21–30 kg/m^2^	242 (59.9)	137 (64.3)	105 (55.0)	
	> 30 kg/m^2^	74 (18.3)	36 (16.9)	38 (19.9)	0.148
Abnormal BMI	< 21 or > 30 kg/m^2^	162 (40.1)	76 (35.7)	86 (45.0)	0.070
Exacerbations previous year^€^	0–1 Exacerbations	243 (70.2)	145 (76.3)	98 (62.8)	
	≥ 2 Exacerbation	103 (29.8)	45 (23.7)	58 (37.2)	< 0.01
Steps per day	< 5000	151 (37.4)	57 (26.8)	94 (49.2)	< 0.01
6MWD predicted	< 70%	203 (50.2)	83 (39.0)	120 (62.8)	< 0.01
MRC^α^	1	99 (26.5)	59 (29.8)	40 (22.9)	
	2	116 (31.1)	72 (36.4)	44 (25.1)	
	3	92 (24.7)	42 (21.2)	50 (28.6)	
	4	42 (11.3)	17 (8.6)	25 (14.3)	
	5	24 (6.4)	8 (4.0)	16 (9.1)	< 0.01
MRC^α^	≥ 3	158 (42.4)	67 (33.8)	91 (52.0)	< 0.01
CIS points^Ω^	Mean (sd)	37.3 (12.5)	36.2 (12.3)	38.7 (12.7)	0.080
CIS^Ω^	≥ 36 points	180 (58.4)	86 (52.8)	94 (64.8)	0.042
Comorbidities					
Arterial hypertension^#^		30 (23.3)	14 (21.5)	16 (25.0)	0.797
Coronary artery disease^#^		9 (7.0)	4 (6.2)	5 (7.8)	0.981
Heart attack^#^		8 (6.2)	4 (6.2)	4 (6.2)	1.000
Heart rhythm disturbance^#^		4 (3.1)	2 (3.1)	2 (3.1)	1.000
Cerebral infarction^#^		6 (4.7)	2 (3.1)	4 (6.2)	0.662
Chronic heart failure^#^		4 (3.1)	2 (3.1)	2 (3.1)	1.000
Peripheral vascular disease^#^		7 (5.4)	3 (4.6)	4 (6.2)	0.983
Anemia^#^		1 (0.8)	0 (0.0)	1 (1.6)	0.994
Diabetes^#^		9 (7.0)	5 (7.7)	4 (6.2)	1.000
Chronic renal failure^#^		3 (2.3)	1 (1.5)	2 (3.1)	0.989
Osteoporosis^#^		8 (6.2)	5 (7.7)	3 (4.7)	0.732
Skeletal muscle dysfunction / muscle weakness^#^		1 (0.8)	0 (0.0)	1 (1.6)	0.994
Arthrosis^#^		13 (10.1)	5 (7.7)	8 (12.5)	0.539
Depression^#^		13 (10.1)	4 (6.2)	9 (14.1)	0.230
Anxiety^#^		7 (5.4)	3 (4.6)	4 (6.2)	0.983
Cognitive impairment^#^		1 (0.8)	1 (1.5)	0 (0.0)	1.000
Gastroesophageal reflux^#^		6 (4.7)	2 (3.1)	4 (6.2)	0.662
Obstructive sleep apnea^#^		2 (1.6)	2 (3.1)	0 (0.0)	0.483
Cancer^#^		4 (3.1)	0 (0.0)	4 (6.2)	0.124
Comorbidity count^#^	0	29 (22.5)	20 (30.8)	9 (14.1)	
	1	48 (37.2)	20 (30.8)	28 (43.8)	
	2 +	52 (40.3)	25 (38.5)	27 (42.2)	0.062

Forced vital capacity (FVC), Forced expiratory volume in one second (FEV_1_), Standard deviation (sd), Interquartile range (IQR), Body mass index (BMI), Medical research council dyspnea score (MRC)

Missing data: ^π^n = 108, ^$^n = 95, ^§^n = 97, ^&^n = 107, ^€^n = 58, ^α^n = 31, ^Ω^n = 96, ^Ⅎ^n = 17, ^∂^n = 23, ^#^n = 275

### Treatable traits and employment status

**Fig. 2:**
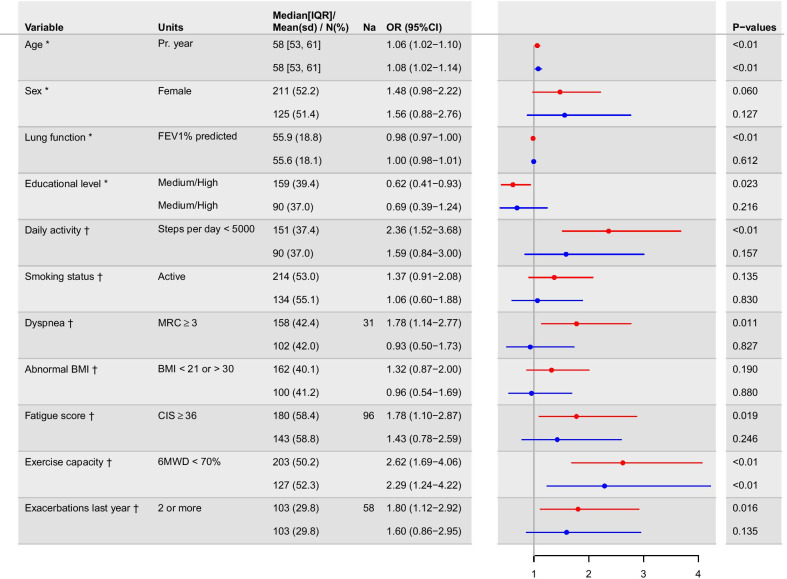
Logistic regression model exploring treatable traits related with not being in paid work. Red model: *OR of model including only confounders. Missing data for specific treatable traits are shown in Na column. †OR of treatable traits adjusted for age, sex, FEV_1_% and educational level. Blue model: Including all confounders * and treatable traits ^†^ in the same model. Due to missing data across different treatable traits 150 patients were excluded from blue analysis leaving 254 with complete data. Odds Ratio (OR), Confidence Interval (CI), standard deviation (sd), Interquartile range (IQR), Forced Expiratory Volume in one second (FEV_1_), Medical Research Council (MRC score), Body Mass Index (BMI), Checklist Individual Strength (CIS), 6 min walk distance (6MWD).

Differences are seen in all treatable traits’ variables favoring patients who are part of the workforce as show in Table 1. Crude unadjusted OR are shown in the appendix for all variables (Additional file 1: Fig. S1). Binomial logistic regression analysis including age, sex, FEV_1_%, and education level revealed significant differences with age, and high education level of not being in paid work and with FEV_1_% predicted increase favoring paid work (see Fig. 2). Analysis of individual treatable traits adjusted for age, sex, FEV_1_, and education level revealed significant differences with daily activity, dyspnea, fatigue, exercise capacity, and exacerbations in the last year of not being in paid work (see Fig. 2). Missing data was present in MRC (N = 31), CIS (N = 96), and exacerbation frequency (N = 58) as shown in Fig. 2.

In the complete model including all treatable traits and confounders, only age and six-minute walk test remained significant. This analysis included 254 patients due to missing data on 150 patients across different variables that were excluded.

## Discussion

### Key findings

In this cross-sectional study of first time referred patients with COPD, below the age of 65, we found approximately half of the patients were not working. Low exercise capacity was the treatable trait that had the highest correlation with not being in paid work as the only treatable trait that remained significant in the fully adjusted model (Fig. 2, Blue model). Low daily activity, higher dyspnea score, higher fatigue score, and two or more exacerbations were however also significantly associated when adjusting for only age, sex, FEV_1_% and education level (Fig. 2, Red model).

### Workforce connection

The percentage of patients outside the workforce in this population (47.3%) is lower than in studies from England (59%) and Denmark (60%) [4, 5]. A reason for this may be that the current study consists of patients referred for the first time to an outpatient respiratory clinic and may therefore exclude patients with more progressive disease who will be more likely to be already on permanent sick leave. An indication of this is seen in the study from UK where 47.2% of patients were in the high age group (60–64) years compared to 35.6% in this study. The lower age in this study may indicate a less vulnerable group which could explain the differences seen in paid work.

### Treatable traits and employment status

The hypothesis that COPD patients without paid work are more vulnerable regarding the prevalence of treatable traits is accepted in this study, with a higher prevalence of all treatable traits in patients without paid work. The causal effects of this relationship are, however, uncertain due to the observational nature of the study. It may be that patients outside the workforce have more treatable traits because they do not have a work life. For example, not being in paid work may enforce a more sedentary lifestyle which leads to a deteriorating health status. It may also be that patients outside the workforce have left the workforce due to an increasing burden of COPD, including lower exercise capacity and daily activity and thereby lost the ability to work. Prospective cohort studies of patients with COPD part of the workforce are needed to explore the causal effects of the associations seen in this study.

The treatable trait most strongly correlated to being outside the workforce is decreased exercise capacity. This variable is the only treatable trait that remains significant (95% CI) in the analysis with all treatable traits and confounders included in the same model (Fig. 2. Blue model). This is consistent with findings in people outside the work force in general and therefore important to incorporate in attempts to improve patients workforce connection [27].

### Possible interventions

Different evidence-based interventions exist for patients with COPD such as pulmonary rehabilitation, smoking cessation, and nutritional advice which aim at preventing decline in function and improve physical activity, exercise capacity and quality of life (QoL) [19, 21, 22, 28]. Whether pulmonary rehabilitation which may improve all the significant treatable traits, will lead to patients returning to work is unknown due to the unknown causal effects of the treatable traits examined [19, 29].

Cross sectional studies have suggested that smoking cessation may improve workforce connection in the COPD population [14, 30]. Smoking status does not reveal significant differences with paid work in our study. This might be while successful smoking cessation may be preceded by a larger burden of tobacco related diseases as a previous study has suggested. Some patients who managed to quit may therefore have higher morbidity than the current smoker and thereby diminish the effect seen in the smoking cessation variable [31].

The finding that patients with higher exacerbation frequency are more likely to be outside the workforce (Fig. 2, Red model), may be related to medicine adherence. Previous studies have found that unemployed patients have poorer medical adherence than patients’ part of the workforce [7]. If medical adherence would have effects on workforce connection is speculative. The negative effects of exacerbation on disease progression however stresses the need for adherence in patients in and especially outside the workforce [32].

This study suggests potential areas of intervention aimed at improving workforce connection. Patients’ part of the workforce could benefit from the interventions by improving and maintaining function hereby preventing workforce detachment. For patients outside the workforce, interventions may help reestablish the workforce connection and improve their general health and QoL. Prospective interventional studies specifically aimed at improving COPD patients’ workforce connection are however lacking and are much needed [33, 34].

Comorbidities were not examined using logistic regression due to much missing data. ANOVA however revealed a tendency towards more comorbidities in patients without paid work (*P* = 0.06 (see Table 1)). This indicates that patients outside the workforce may be more vulnerable with multiple diseases interacting rather than their COPD diagnosis by itself. A relatively high unemployment rate is observed in patients with COPD when compared to other chronic diseases [4]. This makes the attention to patients with COPD especially important when trying to improve workforce connection.

## Limitations

The study has some limitations. First, the prevalence of patients outside the workforce may be different than among other populations because this study only included patients referred for the first time to a secondary clinic. The study is however ideal for evaluating COPD patients’ treatable traits while patients have their first contact with the secondary healthcare system regarding their COPD. Second, knowledge on previous work experience and why patients were not in paid work was not available. It may be that more physical demanding work would be more prevalent among those who have left the workforce. Education level may play a role in this effect but without the data the question remains unanswered in this study. Information on why patients left the workforce may also have confirmed that more females leave the workforce by choice. Third, due to missing data across different variables, the number of patients who could contribute to the analysis of all treatable traits dropped with almost 40% which may also explain why the previously significant variables became non-significant in the full model adjusting for all covariates and traits. The fraction of missing data was evenly distributed across the study sites and we did not have reason to believe that there were systematic recording errors that would bias the findings.

Fourth, pack years may have contributed to the understanding of smoking’s effect on paid work. This was unfortunately not available.

Fifth, we unfortunately did not have access to adherence data as a treatable trait. This information could have improved the understanding of treatable traits and may have helped explain the increased exacerbation frequency seen in patients without paid work.

## Conclusion

Patients with COPD who are not employed have an increased number of treatable traits with exercise capacity being the most important predictor. Interventional studies using for example pulmonary rehabilitation and exercise training addressing individual patient goals related to reestablishing or maintaining workforce connection are needed to explore if workforce connection may be improved.

## Supplementary Information

**Additional file 1: Fig S1.** Logistic regression analysis of unadjusted covariates and treatable traits. Abbreviations: Odds Ratio (OR), Confidence Interval (CI), standard deviation (sd), Interquartile range (IQR), Forced Expiratory Volume in one second (FEV_1_), Medical Research Council (MRC score), Body Mass Index (BMI), Checklist Individual Strength (CIS), 6-min walk distance (6MWD).

## Data Availability

All data generated or analysed during this study are included in this published article [and its supplementary information files].
