# Supplementary material for: Characteristics and treatable traits of patients with chronic obstructive pulmonary disease (COPD) with and without paid employment
Source: Respir Res. 2021 May 12;22:147. doi: 10.1186/s12931-021-01736-6 (PMC8117298; doi:10.1186/s12931-021-01736-6)
Supplement: Supplementary file 1 — Additional file 1: Fig S1. Logistic regression analysis of unadjusted covariates and treatable traits. Abbreviations: Odds Ratio (OR), Confidence Interval (CI), standard deviation (sd), Interquartile range (IQR), Forced Expiratory Volume in one second (FEV1), Medical Research Council (MRC score), Body Mass Index (BMI), Checklist Individual Strength (CIS), 6-min walk distance (6MWD). [file 12931_2021_1736_MOESM1_ESM.pdf]

Variable	Units	Median[IQR]/ Mean(sd) / N(%)	Na	OR (95%CI)	P−values
Age	Pr. year	58 [53, 61]		1.06 (1.02−1.10)	<0.01
Sex	Female	211 (52.2)		1.39 (0.94−2.06)	0.100
Lung function	FEV1% predicted	55.9 (18.8)		0.98 (0.97−0.99)	<0.01
Educational level	Medium/High	159 (39.4)		0.63 (0.42−0.94)	0.023
Daily activity	Steps per day < 5000	151 (37.4)		2.65 (1.75−4.02)	<0.01
Smoking status	Active	214 (53.0)		1.42 (0.96−2.11)	0.078
Dyspnea	MRC ≥ 3	158 (42.4)	31	2.12 (1.39−3.22)	<0.01
Abnormal BMI	BMI < 21 or > 30	162 (40.1)		1.48 (0.99−2.20)	0.056
Fatigue score	CIS ≥ 36	180 (58.4)	96	1.65 (1.04−2.61)	0.032
Exercise capacity	6MWD < 70%	203 (50.2)		2.65 (1.77−3.96)	<0.01
Exacerbations last year	2 or more	103 (29.8)	58	1.91 (1.20−3.04)	<0.01

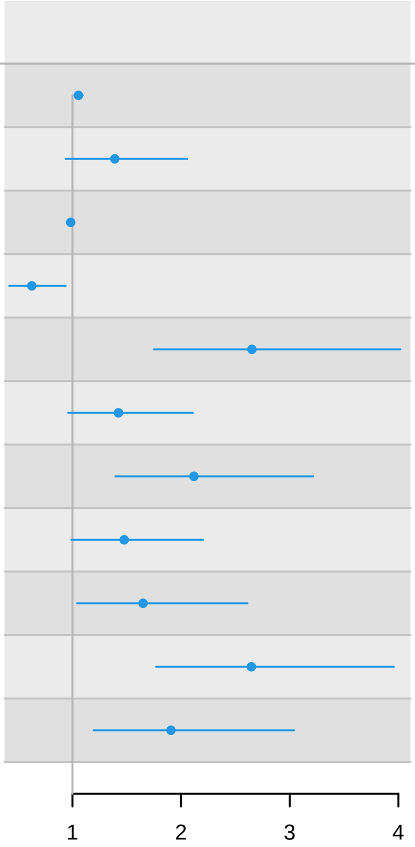

Fig S1. Logistic regression analysis of unadjusted covariates and treatable traits. Abbreviations: Odds Ratio (OR), Confidence Interval (CI), standard deviation (sd), Interquartile range (IQR), Forced Expiratory Volume in one second (FEV1), Medical Research Council (MRC score), Body Mass Index (BMI), Checklist Individual Strength (CIS), 6-minute walk distance (6MWD).
